# Age features of metabolic syndrome and cardiovascular disorders in gout

**DOI:** 10.1186/ar3670

**Published:** 2012-02-09

**Authors:** Surayo Shukurova, Khisrav Toirov, Nabijon Hamidov, Dilfuza Jonnazarova

**Affiliations:** 1 AvicennaTajik State Medical University, Tajikistan

## Materials and methods

Are surveyed 76 gout patients, middle age equaled 56.6 ± 7.5 year. Have been distributed on 3 groups: more younger 50, from 50 to 60 and more senior 60 years. Metabolic syndrome was diagnosed by criteria Adult Treatment Panel III (National Institute of Health, USA) [1]. Serum level of Uric Acid defined by colorimetric enzyme method, glucose - by glucose oxidize method, cholesterol, triglycerides and high density lipoproteides-cholesterol - by colorimetric method [2]. Low and very low density lipoproteides-cholesterol defined by "WT Friedewald Equation" (1972) [3].

## Results

Metabolic syndrome has been diagnosed at 46 (60.5%) patients. Middle age patients with presence of metabolic syndrome has made 55.7 ± 4.7, without - 57.9 ± 8.3 year.

## Conclusions

At the same time we have not revealed age distinctions in occurrence of metabolic syndrome at patients with primary gout, however frequency of IHD of gout patients naturally increased with the years - from 38% to 68%. Patients of the senior age groups the increase in frequency of hypertension and IHD while patients of younger age have obesity, hypertriglyceridemia and hyperglycemia is more often noted.

**Table 1:** Frequency of revealing of signs of metabolic syndrome at gout patients (n = 76)

Sign	Frequency
**CW > 102 cm**	48 (63.2%)
**SBP > 140 mm Hg and/or DBP > 90 mm Hg**	50 (65.8%)
**TG ≥ 120 mg/dl**	22 (29%)
**Glucose ≥ 110 mg/dl**	32 (42.1%)
**HDL-cholesterol < 50 mg/dl**	58 (76.3%)

**CW **- circle waist; **TG **- triglycerides; **SBP **- systolic blood pressure; **DBP **- diastolic blood pressure; **HDL **- high density lipoproteides.

**Table 2:** Frequency of revealing of signs metabolic syndrome at gout patients depending on age, n (%)

**Sign**	**Age groups**		
	
	**<50 y (*n = 26)***	**50-60 y (*n = 26*)**	**>60 y (*n = 24*)**
**CW > 102 cm**	22 (84.6%)	20 (76.9%)	6 (25%)
**SBP > 140 mm Hg and/or DBP > 90 mm Hg**	20 (76.9%)	14 (53.8%)	20 (83.3%)
**TG ≥ 120 mg/dl**	8 (30.8%)	10 (38.45%)	4 (16.7%)
**Glucose ≥ 110 mg/dl**	14 (53.85%)	14 (53.85%)	4 (16.7%)
**HDL-cholesterol < 50 mg/dl**	14 (53.85%)	24 (92.3%)	20 (83.3%)
